# Concomitant Valve Replacement and Coronary Artery Bypass Grafting Surgery: Lessons from the Past, Guidance for the Future? A Mortality Analysis in 294 Patients

**DOI:** 10.3390/jcm13010238

**Published:** 2023-12-30

**Authors:** Kyriakos Spiliopoulos, Dimitrios Magouliotis, Ilias Angelis, John Skoularigis, Bernhard M. Kemkes, Nikolaos S. Salemis, Thanos Athanasiou, Brigitte Gansera, Andrew V. Xanthopoulos

**Affiliations:** 1 Department of Cardiothoracic Surgery, Faculty of Medicine, School of Health Sciences, University of Thessaly, 41500 Larissa, Greece; dmagouliotis@gmail.com (D.M.); t.athanasiou@uth.gr (T.A.); 2 Department of Cardiovascular Surgery, Klinikum Bogenhausen, 81925 Munich, Germanybrigitte_gansera@web.de (B.G.); 3 Department of Cardiology, Faculty of Medicine, School of Health Sciences, University of Thessaly, 41500 Larissa, Greece; iskoular@gmail.com (J.S.); andrewvxanth@gmail.com (A.V.X.); 4 Breast Cancer Surgery Unit, Army General Hospital, 11525 Athens, Greece; nikos.salemis@gmail.com

**Keywords:** valve replacement, coronary artery bypass grafting, mortality analysis, minimal invasive techniques, hybrid procedures

## Abstract

Objective: The aims of this study were to analyze parameters influencing early and late mortality after concomitant valve replacement and coronary artery bypass grafting surgery, using early and long-term information from an institutionally available data registry, and to discuss the results in relation to the current treatment strategies and perspectives. Methods: The study population consisted of 294 patients after combined valve replacement with mechanical prosthesis and CABG surgery. Results: There were 201 men (68.4%) and 93 women (31.6%). Concurrent to the coronary artery bypass grafting, 238 patients (80.9%) underwent aortic-, 46 patients (15.6%) mitral- and 10 patients (3.4%) doublevalve replacement. Cumulative duration of follow up was 1007 patient-years (py) with a maximum of 94 months and was completed in 92.2% (271 cases). Overall hospital mortality (30 days) rate was 6.5% (n = 19). It was significantly higher in patients of female gender, older than 70 y, in those suffering preoperative myocardial infarction, presenting with an additive EuroScore > 8 and being hemodynamically unstable after the operation. Cumulative survival rate at 7.6 y was 78.6%. Determinants of prolonged survival were male gender, age at operation < 70 y, preoperative sinus rhythm, normal renal function, additive EuroScore < 8 and the use of internal thoracic artery for grafting. Subsequent multivariate analysis revealed preoperative atrial fibrillation (HR: 2.1, 95% CI: 0.82–5.44, *p*: 0.01) and risk group of ES > 8 (HR: 3.63, 95% CI: 1.45–9.07, *p* < 0.01) as independent predictors for lower long-term survival. Conclusions: Hospital mortality (30 d) was nearly 2.5-fold higher in female and/or older than 70 y patients. Preoperative atrial fibrillation and/ or a calculated ES > 8 were independent predisposing factors of late mortality for combined VR and CABG surgery. Tailoring the approach, with the employment of the newest techniques and hybrid procedures, to the individual patient clinical profile enables favorable outcomes for concomitant valvular disease and CAD, especially in high-risk patients.

## 1. Introduction

In recent years, numerous studies have reported the early and late results of heart valve replacement (VR) operations in combination with coronary artery bypass grafting (CABG) [1,2,3,4]. The coexistence of valve and coronary artery disease (CAD) poses a challenge for surgical treatment as long as the number of patients suffering from both pathologies, who have been offered cardiac surgery, is constantly increasing. However, risk factors influencing the early and late outcomes of these combined procedures are not clearly defined.

The present study evaluates the determinants of early and late survival of combined VR and CABG by applying uni- and multivariate analysis on available follow-up data from an institutional data registry.

Although all these information and results date back to the period 2001–2007, the findings are compared to the current surgical practice with implementation of less/minimal invasive techniques, as well to future perspectives regarding the therapeutical approach of patients with heart valvular and concomitant coronary artery disease. The discussion with regard to the evolution of cardiac surgery throughout the years aims to emphasize its impact on the outcomes, especially in patients like our collective, who are considered challenging cases of higher risk.

## 2. Materials and Methods

All data were ambispectively collected for 294 consecutive patients undergoing combined VR-CABG at the Krankenhaus München Bogenhausen, Munich, Germany between January 2001 and October 2007. To unify the patient population for study, we included patients who underwent VR with mechanical prosthesis. The exclusion of bioprostheses eliminated any confounding effect they could have on the outcomes. Bioprostheses are, in general, implanted in older, multimorbid patients; have a higher degenerative failure with time; and donot need postoperative anticoagulation treatment, factors that all significantly affect morbidity and mortality. These data are part of a data registry of all cardiac surgical patients at the department. Follow-up data including early and mid- to long-term information were gathered by mail and telephone contact with the patients and/or their referring physicians according to a detailed questionnaire. All information was retrospectively reviewed and analyzed.

### 2.1. Patient Group

Our study group contained 294 patients who underwent combined VR-CABG with mechanical prosthesis. Follow-up was completed in 92.2% (271/294). Twenty-three patients were lost to follow-up, due to a variety of reasons (e.g., change in home address, patient resident in a foreign country, change in referring general practitioner).

There were 201 men (68.4%) and 93 women (31.6%) (mean age: 66.6 y; range: 35.4–86.7 y). Of the total, 238 patients (80.9%) underwent aortic valve replacement (AVR) and coronary artery bypass grafting (AVR-CABG), 46 patients (16.6%) had mitral valve replacement and CABG (MVR-CABG) and 10 patients (3.7%) underwent doublevalve replacement and CABG (DVR-CABG). Demographic and clinical characteristics of the patients are summarized in Table 1.

Left main disease was defined as stenosis greater than 50%. Critical coronary disease was defined as stenosis greater than 70% or more the 60% in the proximal left anterior descending artery.

### 2.2. Operative Technique

In all cases, cardiopulmonary bypass (CPB) was established with moderate hypothermia (32–34 °C). Myocardial protection was achieved with a cold hyperkalemic crystalloid solution (Brettschneider) and external cooling was established with a cold saline solution. Distal anastomoses were constructed first, followed by replacement of valve(s). The approach of the valve was performed by either a transverse aortotomy and/or classical left atriotomy. The proximal anastomoses were performed after closure of the cardiac chambers and, in some cases, on a cross-clamped aorta. The left and right internal thoracic artery (ITA) and saphenous veins were used as conduits for bypass grafting. All patients were admitted postoperatively to the intensive care unit.

### 2.3. Preoperative Variables

Preoperative data included the following: age at operation, gender, associated diseases, New York Heart Association (NYHA) class, valve lesion (stenosis, regurgitation, mixed), left ventricular (LV) ejection fraction (EF), presence of left main stem stenosis, history of myocardial infarction (MI), cardiac rhythm disturbances. Left ventricular function was scored according to the available data of EF and left ventricular end-diastolic pressure (LVEDP).

The European System for Cardiac Operative Risk Evaluation predictive scoring model (EuroSCORE—ES), in both its versions (ESI and ES II),was extensively used and performed well, showing acceptable applicability to different populations of cardiac surgical patients.In light of ES II existing only as a logistic version, scoring patients with continuous non-integer values expressed as percentages, we considered the additive ES with its distinct integer values safer and more reliable for building well-defined, identifiable patient riskgroups. According to the risk predictions derived from the additive ES, and using an arbitrary threshold cutoff point of ES:8, which represents the median value in our study population, the patients were divided into 2 populationally comparable groups (Table 1).

### 2.4. Perioperative Variables

Size of valve prosthesis, site of implant, type of grafts used for bypassing, aortic cross-clamp time were recorded as operative variables. Postoperative complications such as hemodynamic status at the end of surgery, as well death during the hospital stay, were recorded (Table 1).

### 2.5. Outcomes

Perioperative (early) mortality was defined as death before hospital discharge or within 30 days of cardiac surgery regardless of cause. Late mortality was defined as mortality after hospital discharge.

### 2.6. Statistical Methods

Continuous variables are presented as mean ± standard deviation (SD) unless otherwise reported. For univariate analysis of the early mortality, a Pearson Chi-square (χ^2^) test or the Fisher exact test was used to test for differences between groups regarding hospital mortality. Log-linear analysis was applied for the multivariate analysis for hospital mortality including all variables that presented a significant association in the univariate analysis (*p* < 0.05).

Long-term survival estimates were calculated using the method of Kaplan–Meier, whereas univariate comparisons of survival data were tested using the Log-rank and χ^2^ tests. Survival events were analyzedwith regard to gender, age at operation, preoperative clinical status.

For the multivariate analysis of late mortality, the proportional hazard model of Cox was assembled, and predictions were derived.

A *p* value was considered statistically significant when <0.05.

## 3. Results

Cumulative duration of follow-up was 1007 patient-years (py) with a maximum of 94 months.

### 3.1. Early Results

Overall hospital mortality (30 days) rate was 6.5% (n = 19/294).With regard to the type of operation, early mortality rate was, for AVR-CABG, MVR-CABG and DVR-CABG, 5.9%, 8.7% and 10%, respectively (x^2^: 0.47, *p*: 0.7). There were no valve-related early deaths. Eight patients died due to low cardiac output syndrome, five due to multiple organ failure,three due to cardiac arrest and three patients due to other causes (gastrointestinal ischemia, sepsis). Most of the patients (n: 13) died within the first five days after cardiac surgery.

**Figure 1:**
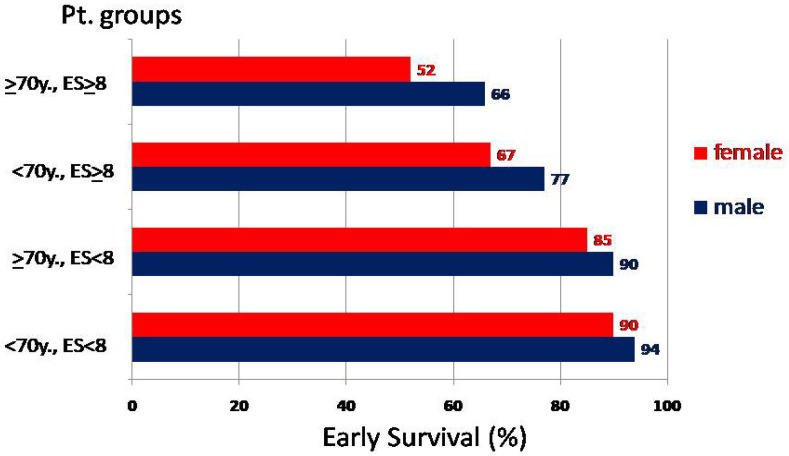
Early survival rates (male vs. female). Abbreviations: Pt: patient; ES: EuroScore; others as previously stated.

Table 2 presents the early mortality rates with regard to the type of operation, age, gender, preoperative NYHA class, presence of left main stem stenosis (LMSST), history of myocardial infarction, aortic cross-clamp time and EuroSCORE (ES) group. In the univariate analysis, significantly (*p* < 0.05) higher hospital mortality presented in females, patients older than 70 y, with MI in their past medical history and patients with a EuroSCORE > 8 (Figure 1). We constructed a statistical model including the above significant calculated clinical parameters and performed a multivariate log-linear analysis. A statistically significant (*p* < 0.05) association between female gender, age at operation ≥70 y, preoperative history of MI, as well ES ≥ 8 and early outcome were identified (Table 3). Especially in females and patients after MI, the early mortality risk was 1.5-fold higher than that of their counterparts.

### 3.2. Late Results

**Figure 2:**
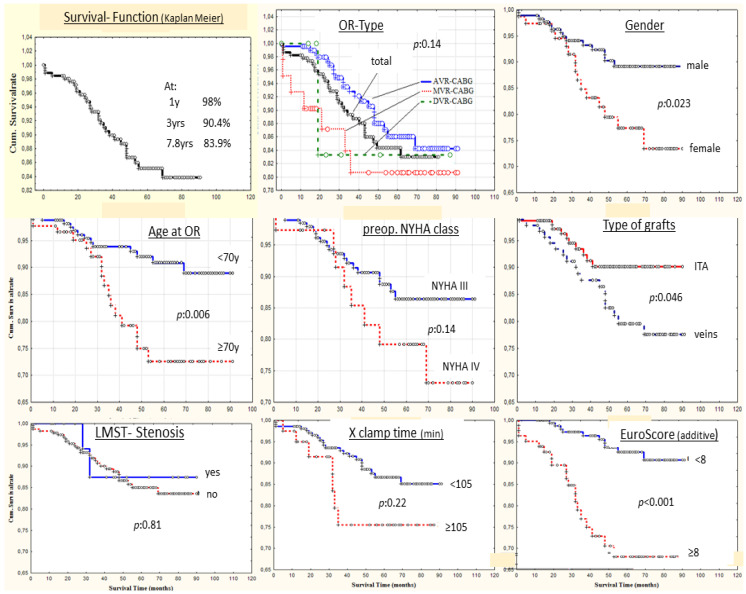
Survival function (Kaplan–Meier). Survival analysis at 94 months (Kaplan–Meier function) evaluating the impact of several variables.Abbreviations. Cum.: cumulative; ITA: internal thoracic artery; LMST: left main stem; others as previously stated.

Hospital survivors were followed during a maximum follow-up period of 94 months. Follow-up was completed in 92.2% (271/294). Late mortality was 11.5% (29/252) with a rate of 2.9%/py at 94 months. The cumulative survival rates at 1, 3 and 7.8 years were 98%, 90.4% and 83.9%, respectively (Figure 2). With regard to the type of operation, the survival rates were, at 7.8 years for AVR-CABG, MVR-CABG and DVR-CABG, 84.5%, 80.6% and 83.8%, respectively (*p*: 0.145) (Figure 2). The same clinical parameters as those tested for hospital mortality were evaluated in univariate analysis for their possible correlation with late death. The type of bypassgrafts was also tested.

Table 4 and Figure 2 illustrate the impact of various variables on the actuarial survival at 94 months. The survival analysis, applying log-rank testing, demonstrated that age at operation ≥70 years, female gender, preoperative impaired renal function, established atrial fibrillation (AF) and higher additive ES (>8) (*p* = 0.015), as well the use only of veins for bypass grafting, were associated with significantly lower survival rates.

A proportional Cox hazard model was assembled for multivariate analysis, including all variables that approached significance in the univariate evaluation. Of the factors tested, AF (HR: 2.1, 95% CI: 0.82–5.44, *p*: 0.01) and risk group of ES > 8 (HR: 3.63, 95% CI: 1.45–9.07, *p* < 0.01) were independently statistically significantly associated with lower long-term survival. Furthermore, females and patients with impaired renal function and supplied only with vein grafts showed an approximately 1.6-, 1.5- and 2-fold, respectively, higher long-term mortality risk compared to their counterparts. However, neither of these factors approached statistical significance in the model (Table 3).

Multivariate analysis for early mortality, applying log-linear analysis of a test model with df (degrees of freedom): 49 at *p*: 0.97 including the following variables: gender, age at operation, preoperative history of myocardial infarction (MI), hemodynamic status at the end of surgery and risk group according to EuroScore.

Cox regression survival analysis (at 94 months) of a test model with df: 6 at *p* < 0.001 and chisquare = 27.85 analyzing following variables: gender, age atsurgery, preoperative renal function and cardiac rhythm, risk group stratified by EuroScore and type of grafts used for bypassing.

## 4. Discussion

Degenerative lesions are the most frequent cause of valve disease in the Western world, and they often occur in older patients, who are also at higher risk for atherosclerotic disease. Particularly, since calcified aortic valve stenosis represents the most common acquired valvular pathology, and as the population ages, the incidence of aortic valve disease with concomitant coronary artery disease is increasing. Patients who undergo combined CABG and AVR surgery are considered at higher operative risk.However, this procedure is currently the third most frequently performed cardiac surgery behind isolated CABG and AVR [5].

Comparing the mortality rates of our study to those of patients undergoing isolated valve replacement with a mechanical prosthesis in our institution, combined procedures of CABG and valve replacement showed a moderate increase in mortality rate of approximately 2.5% [6].

Regarding the still controversial impact of concurrent CABGprocedure at the time of AVR, Formica and co-workers showed in their series that it was not associated with increased mortality in the unadjusted (HR 0.99; 95% CI 0.74–1.33; *p*: 0.99) as well as adjusted analysis (HR 0.83; 95% CI 0.59–1.17; *p*: 0.30). Despite the study limitation of excluding the best and worst cases from the propensity score matching, similar trends were observed in the matched and unmatched survival curves. Thus, the authors advocated that surgical revascularization, firstly, neutralizes the adverse effects of coronary disease; secondly, improves myocardial metabolism and reduces the risk of ischemia in hypertrophied left ventricles; and, subsequently, concluded that CABG combined with AVR should be performed when indicated even in the elderly, without increasing operative risk [7].

The influence of female gender on the outcomes after cardiac operations still remains unclear. Our findings regarding, particularly, the early mortality in correlation with the demographic differences described above (Figure 1), have led us and also other investigators to the proposed hypothesis that hormonal pathways, including abnormalities of the estrogen receptor, ovarian dysfunction, premature menopause and proinflammatory properties of hormone replacement therapy, may be a potentially important cause for increased mortality rates in female patients after cardiac operations [8,9].

The evolution of transarterial aortic valve implantation (TAVI) into a first-choice treatment for high-risk and elderly patients has resulted ina dramatic increase inperformed procedures employing this method in the last decade [10]. Furthermore, in the last years, the indication of TAVI has expanded to nonagenarians [11] and even to intermediate-risk cases [12].

Although the evolution of TAVI offers an alternative in the arsenal of treatment options, the use of lessinvasive methods like TAVI + percutaneous coronary intervention (PCI) compared to the conventional surgical procedure of AVR + CABG remains controversial regarding outcomes. Up to now, only few series have dealt with the issue. Wendt and coauthors [13] presented, in their study comparing, among others, outcomes in high-risk patients after TAVI + PCI and AVR + CABG,an early (30-day) mortality rate of 11.9% and 12.5% (*p* = 0.89), as well survival at 4 years of 53% and 60.7% (*p* = 0.191), respectively. While similar results have been reported in a retrospective study including intermediate-risk patients [14], the recently published prospective randomized SURTAVI trial of intermediate-risk patients undergoing either TAVI + PCI or AVR + CABG revealed favor- and comparable outcomes regarding all-cause mortality and disabling stroke at 2 years with 16% and 14% (*p* = 0.69), respectively [15]. Nevertheless, the current evidence-based established therapeutical approach of isolated TAVI proved to perform better in patients without, compared to those with previous CABG-surgery [16], and to be superior compared to cases after TAVI + PCI [17]. Moreover, in a prospective registry analysis performed by Baumbach et al. comparing characteristics and outcomes of 626 patients, undergoing hybrid procedures of TAVI + off-pumpCABG (OPCAB)/minimally invasive direct CABG (MID-CAB) (OP/MIDCAB), TAVI + PCI, and surgical AVR + CABG, the in-hospital mortality was highest for TAVI + OP/MIDCAB cases (18%), with comparable rates for TAVI + PCI and AVR + CABG groups (9.0 and 6.9%; *p* = 0.009). Mortality at 1 year was more probable after TAVΙ + OP/MIDCAB (HR: 2.17, *p* = 0.002) and TAVI + PCI (HR: 1.63, *p* = 0.010) than after AVR + CABG, with the same holding true forrehospitalization (HR: 2.39, *p* = 0.003 and HR: 1.63, *p* = 0.033). The particularly high in-hospital mortality rate of 18%) in the TAVΙ + OP/MIDCAB group was attributed, on the one hand, to the significantly higher surgical risk (ES 36.4%), disease burden and age of the patients, and on the other,to the potential learning curve of the method, given that the TAVI + OP/MIDCAB hybrid is relatively new and infrequently performed, especially in the case of MIDCAB. These results justified the conclusion that the hybrid treatment of TAVΙ + OP/MIDCAB, despite its slightly poorer long-term outcomes, offers—with TAVI + PCI in patients unsuitable for surgical AVR + CABG—reasonable second-line options [18].

Regarding MV surgery, the issue is more complex due to the variety of pathologies affecting the valve. Our series includes only cases after MV replacement with mechanical prosthesis, without distinguishing between different types of MV disease. While surgery for isolated degenerative MV (DMV) disease is associated with relatively low mortality and acceptable long-term outcomes, especially when reconstructive techniques are performed [1,19,20], patients suffering from ischemic MV (IMV) disease undergoing combined MV-CABG surgery are at higher risk of in-hospital mortality and reduced long-term survival [21].

Although combined MV and CABG surgery has been extensively evaluated in the context of ischemic MV disease, little is known about the early and long-term outcomes of DMV + CABG surgery [2,22].

In a study performed by Bruno et al. including 186 cases who underwent combined DMV + CABG surgery, they showed, in the unmatched as well as matched analysis, a similar rate of mortality, hospital complications, MV repairs and long-term outcomes compared with DMV surgery alone. Concisely, combined surgery showed a similar early mortality compared with DMV-only surgery in the unmatched (6.5% vs. 5.4%, *p*: 0.71) and matched analysis (7.5% vs. 8.2%, *p*: 0.82), while the 10-year survival rate was 70.5% vs. 68.6% (*p*: 0.07) for the unmatched and 64.6% vs. 62.5% (*p*: 0.9) for the matched analysis, respectively. Mitral valve repair had a beneficial effect on short-term outcomes and long-term mortality rates, regardless the presence of concomitant coronary surgery [23]. Although CABG was not an independent predictor of adverse outcomein this study, this was not the case in a largeUS series reported by Vassileva et al. [1]. However, the last one consisted of an older patient cohort encompassing various valve pathologies and surgical techniques. Conclusively, in the context of concomitant severe DMV and coronary artery disease, the preferred treatment is combined DMV + CABG surgery instead of alternative and less validated options. Additionally, MVrepair provided superior short-term health outcomes and long-term survival [23].

Concerning ischemic mitral regurgitation (IMR), asidefrom the cornerstone of surgical treatment through CABG surgery for coronary artery disease, most agree that severe MR requires either surgical valve replacement or repair. However, for a moderate degree of valve insufficiency, the surgical approach remains controversial. Up to now, there are no data available from randomized controlled trials proving the safety of the combined surgical procedure, and results from observational studies are inconsistent.

Percutaneous approaches to the treatment of multivessel CAD and MR offer alternative therapeutic options in specific subsets of patients that are athigh risk fromundergoing the established traditional surgical procedures. While coronary artery disease (CAD) is addressed usingthree techniques,namely,percutaneous coronary intervention (PCI), hybrid coronary revascularization (HCR) seeking tocombine the advantages of CABG and PCI, and conventional CABG (including MIDCAB), MR is treated beside the accepted traditional sternotomy through less invasive approaches like percutaneous edge-to-edge repair (PEER) with positioning and deployment of a clip fixating the edges of the mitral leaflets aiming to decrease regurgitant flow, and minimal incisions via mini right thoracotomy (mini-MVR). Theoretically, IMR and CAD treatment options include atotal of nine possible approaches: CABG + MVR, CABG + mini-MVR, CABG + PEER, HCR + MVR, HCR + mini-MVR, HCR + PEER, PCI + MVR, PCI + mini-MVR, and PCI + PEER. Of those, HCR + MVR and CABG + mini-MVR are not reasonable. While the procedure of CABG + MVR represents the golden standard of care, the remaining six approaches (CABG + PEER, HCR + mini-MVR, HCR + PEER, PCI + MVR, PCI + mini-MVR, and PCI + PEER) have various attributes that potentially offer better treatment options to subsets of IMR cases in whom the current standard of care is of high risk [24].

Concerning current evidence in the topic, there is a lack of large series analyzing the outcomes of these approaches. However, some groups have evaluated hybrid PCI procedures combined with valve operations. In this context,in 2012, Santana et al. published their experience of over 200 patients undergoing PCI for coronary revascularization combined with a minimally invasive valve procedure. They presented early mortality and all-cause mortality rates at 4.5 years of 3.6% and 12%, respectively. Additionally, hybrid patients, compared to those who underwent conventional sternotomy, showed lower complication rates and lengths of stay [25].

In a recent study by George et al. including 26 cases who underwent a single-stage hybrid PCI procedure of a non-LAD vessel followed by a valve operation, there were risk reductions of 35% and 17% in the re-operative and non-re-operative patient cohorts, respectively, when recalculating the STS risk after the PCI was performed [26].

In general, there are some concerns regarding the combined approach of a PCI and valve operation. Despite the lower morbidity and mortality rates associated with a hybrid approach, a major drawback of the concomitant PCI and valve procedure is an increased incidence of acute kidney injury; thus, a staged procedure is recommended, with a period of three weeks between both interventions [27]. On the other hand, if PCI is performedprior to surgery, there is a potential increased bleeding risk because of the antiplatelet treatment with clopidogrel [28]. However, latter seems to not be significant [29].

## 5. Conclusions

The findings of our analysis provide clinical insights into what historically represent some of the most challenging, in every aspect, procedures in heart surgery. Independent predisposing factors for impaired early and long-term outcomes after concomitant valve replacement and coronary artery bypass grafting surgery were female gender, advanced age (>70 y) at operation and preoperative atrial fibrillation, as well as a calculated ES > 8, respectively. However, tremendous innovations in surgical techniques and devices, as well as theconstant evolution in patient care, have resulted in improved outcomesin recent years. The employment of minimally invasive surgical approaches and/or percutaneous transcatheter techniques to the individual patient clinical profile enables favorable outcomes, especially in certain subsets of high-risk patients that are unable to undergo conventional heart surgery. Nevertheless, these procedures have yet to be further evaluated through large prospective randomized trials.

## 6. Limitations

This retrospective nonrandomized series refers to a single-center regional experience; thus, the results may not be generalizable to the entire population, since there are significant differences between institutions and countries. Additionally, its relatively small size of 294 cases may be another source of bias in the statistical analysis of the results. Furthermore, the relatively high percentage of 8% of missing long-term follow up data, and the relatively low proportions of female patients (31%), DVR-CABG (3.4%) and MVR-CABG (15.6%) procedures result inan inhomogeneous collective influencing the evaluation.

## Figures and Tables

**Table 1:** Demographic and clinical characteristics of the patients.

Variable	AVR-CABG	MVR-CABG	DVR-CABG	Total
	n: 238	n: 46	n: 10	n: 294
Patient related				
Gender:				
Male	175 (73.5%)	22 (47.8%)	4 (40%)	201 (68.4%)
Female	63 (26.5%)	24 (52.2%)	6 (60%)	93 (31.6%)
Age (y):				
Range	35.4–86.7	45–85.9	56.7–76.6	35.4–86.7
Mean	66.16	67.8	70.7	66.6
LV-EF:				
>50%	146 (61.3%)	27 (58.7%)	7 (70%)	180 (61.2%)
30% < x ≤ 50%	77 (32.3%)	16 (34.8%)	3 (30%)	96 (32.6%)
≤30%	15 (6.4%)	3 (6.5%)	0 (0%)	18 (6.2%)
Preop. NYHA:				
I/II	2/14 (6.7%)	0/0 (0%)	0/0 (0%)	16 (5.5%)
III/IV	188/34 (93.3%)	34/12 (100%)	9/1 (100%)	278 (94.5%)
EuroScore (additive):				
Range	2–19	2–15	3–10	2–19
Mean ± SD	5.98 ± 3.17	8 ± 3.19	7 ± 2.26	6.86 ± 2.95
Valve lesion:				
Stenosis	156 (65.5%)	4 (8.7%)	2 (20%)	162 (55.1%)
Regurgitation	22 (9.25%)	30 (65.2%)	1 (10%)	53 (18%)
Mixed	60 (25.25%)	12 (26.1%)	7 (70%)	79 (26.9%)
Comorbidity:				
Diabetes mellitus	67 (28.15%)	13 (28.3%)	2 (20%)	82 (27.9%)
Renal failure	3 (1.3%)	0 (0%)	0 (0%)	3 (1%)
Art. hypertension	150 (63%)	26 (56.6%)	7 (70%)	183 (62.2%)
PVD	44 (18.5%)	6 (13.05%)	1 (10%)	51 (17.3%)
CVD	20 (8.4%)	3 (6.52%)	0 (0%)	23 (7.8%)
Left main stem stenosis	19 (8%)	6 (13.05%)	0 (0%)	25 (8.5%)
Myocardial infarction:				
<48 h	3 (1.3%)	3 (6.5%)	0 (0%)	6 (2%)
<3 weeks	1 (0.4%)	1 (2.2%)	1 (10%)	3 (1%)
>3 weeks	72 (30.3%)	22 (47.8%)	0 (0%)	94 (32%)
Cardiac rhythm:				
Sinus	195 (81.9%)	28 (60.9%)	7 (70%)	230 (78.2%)
Atrial fibrillation	31 (13%)	14 (30.5%)	2 (20%)	47 (16%)
Other	12 (5.1%)	4 (8.6%)	1 (10%)	17 (5.8%)
Operation related				
Time (min):				
CPB (mean)	53–241 (107)	71–455 (133)	94–624 (177)	53–624 (114)
X-clamp (mean)	41–207 (84.5)	56–322 (100)	80–229 (117)	41–322 (88)
Operation (mean)	90–400 (189)	120–530 (222)	160–660 (245)	90–660 (196)
Grafts: range (/pt):	1–5 (2.03/pt)	1–4 (2.04/pt)	1–3 (1.4/pt)	1–5 (2.01/pt)
bITA	31 (13%)	5 (11%)	1 (10%)	37 (12.6%)
sITA	129 (54%)	16 (35%)	6 (60%)	151 (51.4%)
Veins	78 (33%)	25 (54%)	3 (30%)	106 (36%)
Comp. revascularization	226 (95%)	44 (95.6%)	10 (100%)	280 (95.2%)
Size of impl. prosthesis:			AV MV	
19/21 mm	12/56 (28.5%)	0/0 (0%)	1/3 0	13/59
23/25 mm	101/52 (64.5%)	1/22 (50%)	4/2 1/2	107/78
27/29 mm	17/0 (7%)	21/2 (50%)	0/0 3/2	41/4
Status at OR-end:				
Stable without inotropes	104 (43.7%)	12 (26.09%)	4 (40%)	120 (40.81%)
Low inotropes	126 (52.94%)	24 (52.18%)	5 (50%)	155 (52.72%)
High inotropes/IABP	8 (3.36%)	10 (21.73%)	1 (10%)	19 (6.47%)

Abbreviations: AVR: aortic valve replacement; MVR: mitral valve replacement; DVR: double valve replacement; CABG: coronary artery bypass grafting; n: number; y: years; LV-EF: left ventricular ejection fraction; preop.: preoperative; NYHA: New York Heart Association; SD: standard deviation; Art.: arterial; PVD: peripheral vascular disease; CVD: central vascular disease; h: hours; min: minutes; CPB: cardiopulmonary bypass; X-clamp: cross-clamping; pt: patient; bITA: bilateral internal thoracic artery; sITA: single internal thoracic artery; Comp.: complete; impl.: implanted; AV: aortic valve; MV: mitral valve; mm: millimeters; OR: operation; IABP: intra-aortic balloon pump.

**Table 2:** Early mortality rates.

**Variable**	**Early Mortality (%)**	**χ^2^**	***p***
Patient related:			
Age at OR (<70 y./≥70 y.)	4.23/10.48	4.35	0.037
Gender (male/female)	3.48/12.9	9.33	0.0023
preop. LV-EF (<50%/≥50%)	7.96/5.52	0.69	0.4
preop. NYHA class (I, II/III, IV)	6.25/6.47	<0.0001	0.97
Left main stem stenosis (yes/no)	8/6.32	0.11	0.74
preop. Myocardial Infarction (yes/no)	11.65/3.66	7.06	0.0079
Renal failure (yes/no)	12.2/5.75	1.97	0.16
Diabetes mellitus (yes/no)	7.31/6.13	0.14	0.71
Arterial hypertension (yes/no)	6.55/6.31	0.01	0.93
Cardiac rhythm (SR/AF)	5.22/6.38	0.1	0.75
EuroScore (additive) (<8/≥8)	2.86/14.58	13.07	<0.001
Operation related:			
Type of OR (AVR-CABG/MVR-CABG/DVR-CABG)	5.88/8.7/10	0.7	0.4
Xclamp time (<83 min/≥83 min)	5.48/7.43	0.46	0.49
(<105 min/≥105 min)	5.48/9.33	1.37	0.24
Complete revascularization (yes/no)	6.8/0	1.02	0.31
Status at OR-end (stable/inotropes)	3.63/47.36	56.23	<0.001
**Variable (Survivors/Non-Survivors)**	**Mean**	***p***	
Age at OR (y.)	66.17/72.43	<0.001	
X-clamp time (min)	86.93/107.73	0.004	
EuroScore additive	6.64/9.78	<0.001	
No of bypass grafts	2.01/2.05	0.86	

Abbreviations: χ^2^: chi square; *p*: *p* value; SR: sinus rhythm; AF: atrial fibrillation; No: number; others as previously stated.

**Table 3:** Multivariate analysis for early mortality and long-term survival (94 months).

Log-Linear Analysis (Early Mortality)Variable	χ^2^		*p*
Gender	8.38		0.003
Age at OR	4.34		0.003
Status post-MI preop.	6.65		0.01
Status at end of OR	1.04		0.3
EuroSCORE	10.94		<0.001
Cox Regression Survival Analysis (94 mo.)Variable	HR	95% CI	*p*
Gender (female)	1.604	0.699–3.679	0.263
Age at OR (≥70 y.)	0.882	0.345–2.251	0.793
Renal function (impaired)	1.496	0.561–3.993	0.420
Cardiac rhythm (AF)	2.122	0.827–5.440	0.011
EuroSCORE (>8)	3.637	1.457–9.075	0.005
Type of grafts (veins)	1.99	0.832–4.772	0.121

Abbreviations: as previously stated.

**Table 4:** Univariate analysis evaluating the impact of various variables on survival at 94 months.

Variable	Survival at 94 mo. (%)	Log-Rank Test *p*
Patient related:		
Age at OR (<70 y./≥70 y.)	89.1/73.1	0.006
Gender (male/female)	89.15/74.6	0.023
Preop. LV-EF (35% ≤ x < 50%/≥50%)	84.45/84.6	0.82
Preop. NYHA class (III/IV)	86.6/74.7	0.14
Left main stem stenosis (yes/no)	87.2/83.8	0.81
Preop. myocardial Infarction (yes/no)	83.9/84.3	0.62
Renal failure (yes/no)	67.9/86.5	0.03
Diabetes mellitus (yes/no)	76.4/86.3	0.068
Arterial hypertension (yes/no)	82.4/86.7	0.25
Cardiac rhythm (SR/AF)	87.75/75.4	0.022
ES (additive) (<8/≥8)	90.9/68.65	<0.001
Operation related:		
Type of OR (AVR-CABG/MVR-CABG/DVR-CABG)	84.45/80.7/86.7	0.14
X-clamp time (<83 min/≥83 min)	84.4/83.5	0.71
(<105 min/≥105 min)	85.0/80.1	0.22
Complete revascularization (yes/no)	84.3/76.7	0.66
Grafts (ITA/vein)	90.2/77.8	0.046
(sITA/bITA/vein)	98.3/89.1/77.8	0.17
Status at OR-end (stable/inotropes)	86.2/82.2	0.49

Abbreviations: AVR: aortic valve replacement; MVR: mitral valve replacement; DVR: double valve replacement; CABG: coronary artery bypass grafting; y: years; LV-EF: left ventricular ejection fraction; preop.: preoperative; NYHA: New York Heart Association; min: minutes; X-clamp: cross-clamping; bITA: bilateral internal thoracic artery; sITA: single internal thoracic artery; OR: operation.

## Data Availability

The data that support the findings of this study are available from the corresponding author upon reasonable request.
